# The Use of the ICF Classification Sheet to Assess Cognitive-Behavioral Disorders and Verbal Communication in Patients after Ischemic and Hemorrhagic Stroke during Rehabilitation

**DOI:** 10.3390/ijerph191912127

**Published:** 2022-09-25

**Authors:** Ewa Lucka, Mateusz Lucki, Marcin Cybulski, Przemysław Daroszewski, Przemysław Lisiński

**Affiliations:** 1 Department of Rehabilitation and Physiotherapy, University of Medical Sciences, 28 Czerwca 1956 Str., No 135/147, 60-545 Poznań, Poland; 2 Department of Clinical Psychology, University of Medical Sciences, Bukowska 70 Str., 60-812 Poznań, Poland; 3 Department of Organization and Management in Healthcare, University of Medical Sciences, 60-545 Poznań, Poland

**Keywords:** ICF, stroke, cognitive disorders, behavioral disorders, verbal communications

## Abstract

*Background:* In patients after experiencing stroke, the cognitive-behavioral deficits and disorders of verbal communication limit the effectiveness of rehabilitation. The key is to diagnose them at an early stage of rehabilitation and to implement appropriate psychological and speech therapy. *Objective:* Identify differences in the frequency and effectiveness of cognitive-behavioral disorder therapy depending on the clinical type of stroke, assessed before and after rehabilitation treatment, and their presentation using the ICF (International Classification of Functioning, Disability, and Health) classification. *Materials and Methods:* The study was prospective and included the analysis of cognitive-behavioral and verbal communication disorders. The study consisted of 47 patients after intracerebral hemorrhage (ICH) and 47 patients after an ischemic stroke (IS) before the implementation of rehabilitation and after completing a 4-week rehabilitation. *Results:* In the group after ICH, psychological therapy significantly reduced the disturbances of consciousness and orientation (*p* < 0.001) and improved the speed of performing tasks in tests (*p* < 0.001). In patients after IS and ICH, memory and attention function improved significantly (*p* < 0.001). Moreover, in patients after ICH, language function deficits decreased significantly (*p* = 0.018). Mood disturbances were maintained in 17% of patients after ICH and 40% of patients after IS (*p* = 0.007). Speech therapy reduced speech articulation disorders and aphasia in 85% of patients after ICH (*p* = 0.001) and in 68% of patients after IS (*p* = 0.033). *Conclusions:* The frequency and type of cognitive-behavioral and verbal communication disorders vary depending on the history of ICH or IS. The ICF classification may be useful in assessing and analyzing cognitive-behavioral and verbal communication disorders, which may lead to the implementation of appropriate psychological and speech therapy at an early stage of rehabilitation and increase the effectiveness of the therapy.

## 1. Introduction

The clinical consequences of stroke constitute the third leading cause of mortality and the second leading cause of long-term disability in Poland [1,2]. Stroke disability is multifactorial, with a wide variety of clinical symptoms; it differs depending on the neurological state, location and type of stroke [3]. The cognitive-behavioral deficits and disorders of verbal communication limit the effectiveness of rehabilitation in patients after stroke. Cognitive-behavioral disorders are observed in 40–70% of stroke patients. Failure to recognize these disorders and, consequently, failure to implement targeted therapy increases the degree of disability and mortality [4]. At three months and 1, 2 and 3 years post-stroke, the prevalence rates of cognitive-behavioral deficits were 39%, 35%, 30% and 32%, respectively [5]. Post-stroke cognitive impairment resulting from ischemic stroke (IS) or hemorrhagic stroke (ICH) is the main source of post-stroke mortality worldwide [6]. Understanding the complex interaction between stroke type and functional status remains a priority and is critical to the development of targeted rehabilitation. Information on the actual experiences of patients is necessary to create and implement rehabilitation programs and to identify the needs of people with disabilities [7]. The therapy of cognitive dysfunctions in combination with speech therapy in patients after stroke significantly reduces disorders of speech articulation and interpersonal communication [8].

The ICD-10 (International Statistical Classification of Diseases and Related Health Problems) includes causes of stroke. According to the ICD-10 classification, the diagnosis does not provide sufficient information on the functional status and impairment of fitness and independence. Due to pathophysiological differences, IS and ICH have different long-term cerebral and functional consequences [9]. The attempt to standardize the rehabilitation protocol has led to the creation of a universal tool that unambiguously describes the patient’s condition in terms of damage, dysfunction, and activity, which allows for the comparison of results obtained with the same tools while enabling the data to be recorded in electronic form [10,11]. The described conditions are met by the International Classification of Functioning, Disability, and Health (ICF). The effect of implementing ICF in clinical practice is coding the state of health and disability with the possibility of comparing the functional condition over time in a specific patient in a group of people suffering from the same disease [12].

In the available literature, the ICF classification was used to recode data contained in the medical records of patients after stroke [13,14], and the correlation of the ICF classification with commonly used methods of clinical assessment of stroke in acute and chronic rehabilitation was also assessed [15]. However, for patients after IS and ICH, the results of the ICF classification category for assessing cognitive-behavioral disorders and verbal communication during rehabilitation have not been presented so far. The main goal of our study was to try to answer the question of how the type of stroke determines the disorders of cognitive and verbal communication disorders. The specific aim of the study was to use the ICF classification category as a tool to present differences in the occurrence of psychotherapeutic and speech therapy disorders in patients after IS and ICH before and after rehabilitation treatment.

## 2. Materials and Methods

### 2.1. Study Group/Inclusion and Exclusion Criteria

The study was prospective. The principles of ethical biomedical research set out in the Declaration of Helsinki were respected. The condition for qualifying for the study was a stable clinical condition and consent to participate. The study included an analysis of cognitive-behavioral and verbal communication disorders based on a clinical examination performed by a psychologist and speech therapist on admission to the Neurological Rehabilitation Department of the Rehabilitation Clinic at the Orthopedic and Rehabilitation Clinical Hospital Wiktor Dega in Poznań before the implementation of rehabilitation treatment and again after its completion on the day of discharge from the Department in the period from December 2017 to September 2020. Patients were divided into two groups depending on the IS or ICH history. As part of the rehabilitation program, each patient underwent psychological therapy for cognitive-behavioral disorders and speech therapy for language deficits. The therapy was conducted daily and included one hours of personal psychological therapy, one hours of individual speech therapy and one hours of group therapy focused on interpersonal communication.

Personal psychological therapy was individualized in terms of the patient’s needs and was based on:

1. Restoring executive functions (e.g., day planning training, the chronology of proceedings, supervision over the implementation of daily duties).
2. Training of memory processes (e.g., short-term memory in the verbal and visual modality and long-term memory in information encoding and decoding).
3. Attention processes training (focusing attention on one indicated stimulus and shifting and dividing attention between several stimuli).

For this purpose, sets of paper-pencil exercises (worksheets for exercises in cognitive and executive functions) were used. Additionally, Poppelreuter Tables (Poppelreuter Tables) and open source PEBL (Psychology Experiment Building Language) software. Mackworth Clock Test, Time Wall and Connections. Despite the experimental and, above all, diagnostic nature of the tools, in practice, it has training possibilities. Additionally, elements of Solution Focused Brief Therapy and mindfulness training were used as supportive.

Personal speech therapy was based on exercises covering the essential linguistic functions: creating and understanding linguistic statements, reading, and writing. Additionally, the therapy was conducted using the AfaSystem computer program, extended package version 2.1.1.1 dated 16 July 2017, Harpo (Poznań, Poland). The AfaSystem program consists of a management system (patient catalog, preparation of exercise sets) and thirty-two therapeutic programs (modules). Speech therapies are organized according to the disorders of the linguistic function, based on which tasks of understanding linguistic messages are created. The program includes duties based on illustrations and audio presentations sorted by difficulty levels, among others, according to frequency and word complexity. During therapy, a speech therapist has many modifications that allow individual adjustment of tasks to the current needs and capabilities of a particular patient.

The language message comprehension therapy included the differentiation of the wording (auditory analysis of linguistic stimuli), the interpretation of the meaning of the messages (understanding linguistic content of varying complexity), and the use of verbs (understanding the audio content related to an activity).

Linguistic expression therapy included forming statements based on the development of nouns, adjectives, phraseological compounds, and the updating of names in sentences.

Reading therapy included: reading nouns, verbs, adjectives, synonyms, reading sentences (developing nouns in context), arranging sentences (building complex statements), developing comparisons in context (reading sentences) and out of context (matching words to a pair), abstract thinking by developing nouns based on phraseological compounds.

Writing therapy included: writing nouns, verbs, adjectives, antonyms, and writing phraseological relationships with clarification of meaning, writing compound sentences.

The study inclusion criteria were as follows: (1) patients after the first episode of IS or ICH (2) confirmed stroke by medical imaging (3) hospitalized patients in the early period after stroke (3) up to 14 days from the diagnosis of stroke, in the neurological rehabilitation department, (4) complete medical records regarding the assessment of cognitive disorders and verbal communication made by a psychologist and speech therapist on admission and discharge from the Department (5) assessment of the patient’s condition in the NIHSS (National Institutes of Health Stroke Scale) below 12 points (6) completion of 4-week psychological therapy and speech therapy in the early period after stroke.

The study exclusion criteria were as follows: (1) patients with multiple histories of strokes (2) lack of imaging studies in the medical records confirming the occurrence of a stroke (3) patients hospitalized as part of late neurological rehabilitation, more than 14 days after the diagnosis of stroke, in the neurological rehabilitation department (4) patients with whom it was impossible to establish contact and obtain consent to participate in study (5) patients who did not undergo a full assessment of cognitive disorders and verbal communication by a psychologist and speech therapist (6) an NIHSS stroke score above 13 points (7) discontinuation of psychological therapy and/or speech therapy during rehabilitation. After applying the inclusion and exclusion criteria, 47 patients with a history of ICH and 47 with a history of IS were finally qualified for the study. All patients underwent a 4-week early neurological rehabilitation focused on psychological and speech therapy for cognitive-behavioral disorders and verbal communication. The results were recoded into ICF classification categories and presented in a graphical form.

The Bioethics Committee approved the study of the Karol Marcinkowski University of Medical Sciences in Poznań No. 810/2017 of 22 June 2017.

### 2.2. Cognitive-Behavioral Disorders

The assessment was made on the basis of a standardized hospital psychological examination protocol. Before the implementation of rehabilitation, each patient had an examination qualifying for psychological therapy. The examination was also repeated after completed the rehabilitation. The disorders of consciousness and allopsychic orientation, autopsychic orientation, attention and memory functions, emotional-personality system, executive functions, agnosia and apraxia were analyzed. Qualitative and quantitative research was carried out based on the observations and interview of a clinical psychologist experienced in working with stroke patients. The qualitatively assessed types of dysfunction and the depth of disturbances in the discussed areas were described in intervals on a scale from 0 to 4, where 0—means no disturbances, 1—slight functional disturbance but not significantly influencing the functioning in daily living, 2—small disturbances of functions affecting functioning in daily living, 3—moderate function disturbances that interfere with daily living, 4—severe disturbances that prevent independent functioning in daily living.

### 2.3. Verbal Communication Disorders

Before the implementation and after the completion of rehabilitation, each patient underwent an examination qualifying them for speech therapy. The same neurologist carried out the research. The assessment was made based on a standardized hospital protocol of speech therapy examination, which included an analysis of the depth of speech, language, and interpersonal communication disorders. To describe the depth of these deficits, a scale from 0 to 4 was used, where 0—no disturbances, 1—slight functional disturbances but not significantly affecting functioning in daily living, 2—slight functional disturbances affecting functioning daily living, moderate dependence by supervision or belaying, 3—disturbance of functions of moderate intensity, clearly disrupting daily living, assistance required 4—disturbance of functions to a severe degree, preventing independent functioning in daily living, complete dependence.

### 2.4. ICF Profile

To better emphasize the differences in the type and frequency of cognitive-behavioral and speech therapy disorders in the analyzed clinical types of stroke, the graph presents the percentage distribution of the category qualifiers according to the ICF classification markings: qualifier 0—no disorder present if the percentage distribution was from 0% to 4%, taken as dark green; 1—insignificant occurrence of a disturbance if the value of the percentage distribution was from 5% to 24%, the light green color was used; qualifier 2—moderate occurrence of the disorder if the value of the percentage distribution was from 25% to 49%, the yellow color was used; qualifier 3—significant occurrence of the disorder if the value of the percentage distribution was from 50% to 95%, the orange color was used; qualifier 4—extremely high occurrence of disturbance—if the value of the percentage distribution was from 96% to 100%, the red color was used.

#### 2.4.1. ICF Categories of Cognitive Behavioral Disorders

According to the ICF classification, the following codes and qualifiers have been assigned to the individual categories of psychological disorders:

Disturbances of consciousness were coded in category b110 consciousness functions and the following qualifiers were assigned: qualifier 0—full consciousness, qualifier 1—sleepy, qualifier 2—not fully conscious, qualifier 3—unconscious

Disorientation was coded in category b114 orientation functions and the following qualifiers were assigned: qualifier 0—full orientation as to time and place preserved, qualifier 1—orientation to place and time preserved, qualifier 2—lack of full orientation as to the place or time, qualifier 3—complete disorientation as to place and time

Mood disorders were coded in category b130 energy and drive functions, and the following qualifiers were assigned: qualifier 0—no mood disorders, qualifier 1—depressed mood.

Attention system disorders were coded in category b140 attention functions and the following qualifiers were assigned: qualifier 0—no disturbances in attention systems, qualifier 1—slight attention disorders, qualifier 2—moderate attention disorders, qualifier 3—severe attention disorders.

Memory disorders were coded in category b144 memory functions and the following qualifiers were assigned: qualifier 0—no memory impairment, qualifier 1—mild memory impairment, qualifier 2—moderate memory impairment, qualifier 3—severe memory impairment.

Psychomotor slowing down was coded in category b147 psychomotor functions and the following qualifiers were assigned: qualifier 0—no psychomotor slowing down, qualifier 1—psychomotor slowing down.

Emotional disorders were coded in category b152 emotional functions and the following qualifiers were assigned: qualifier 0—no emotional disturbances, qualifier 1—slight emotional disturbances, qualifier 2—moderate emotional disturbances, qualifier 3—severe emotional disturbances.

Agnosia was coded in category b156 perceptual functions and the following qualifiers were assigned: qualifier 0—no agnosia 1—light agnosia, qualifier 2—moderate agnosia, 3—severe agnosia.

Executive system disorders were coded in category b164 higher cognitive functions and the following qualifiers were assigned: qualifier 0—no disorders of the executive systems, qualifier 1—slight disorders of the executive systems, qualifier 2—moderate disorders of the executive systems, qualifier 3—severe disorders of the executive systems.

Apraxia was coded in category b176 mental functions of complex movements and the following qualifiers were assigned: qualifier 0—no apraxia, qualifier 1—slight apraxia, qualifier 2—moderate apraxia, qualifier 3—severe apraxia.

#### 2.4.2. ICF Categories of Verbal Communication Disorders

According to the ICF classification, the following codes and qualifiers have been assigned to individual categories of speech therapy disorders:

Language disorders were coded in category b167 mental functions of language and the following qualifiers were assigned: qualifier 0—linguistic functions preserved, qualifier 1—partially impaired language functions, qualifier 2—completely impaired linguistic functions.

Speech fluency was coded in category b1671 expression of language and the following qualifiers were assigned: qualifier 0—no speech disorders, qualifier 1—blurred speech, qualifier 2—dysarthric speech.

Aphasia was coded in category b1672 integrative language functions and the following qualifiers were assigned: qualifier 0—no aphasia, qualifier 1—sensory or motor aphasia, qualifier 2—sensory and motor aphasia

Dysphagia was coded in category b5108 ingestion functions and the following qualifiers were assigned: qualifier 0—no dysphagia qualifier 1—dysphagia.

Speech disturbances were coded in the category speaking d330 and the following qualifiers were assigned: qualifier 0—no word choice disorders, qualifier 1—inadequate, limited word choice, qualifier 2—no sentence formulation, qualifier 3—no speech, making sounds.

Social communication disorders were coded in category d710 basic interpersonal interactions and the following qualifiers were assigned: qualifier 0—preserved, qualifier 1—moderately disturbed, qualifier 2—completely disturbed.

### 2.5. Statistical Analysis

Statistica version 13.1 TIBICO Software Int. (Kraków, Poland), and MS Excel from Microsoft Office 2019 MSO, version 2208, (Redmond, WA, USA) were used to elaborate the results. Descriptive statistics were presented using: mean, standard deviation (SD), median, minimum and maximum. In the case of qualitative variables, the number of people in each category was described and the percentages were calculated. The normality of the distribution was checked with the Shapiro–Wilk test, and the homogeneity of variance with the Levene’s test. Depending on the nature of the variables and the fulfilled assumptions, parametric or non-parametric tests were used, respectively. The student’s *t*-test for independent samples or the non-parametric Mann–Whitney U test was used to check whether there were significant differences between the results obtained by people after ischemic and hemorrhagic stroke. Paired student’s *t*-test or non-parametric Wilcoxon pairwise test were used to compare pre-treatment and post-treatment results within each group. The results expressed in the nominal scale were dependent on the Pearson chi² test or the Fisher test, while changes in the values of these parameters occurring after treatment were assessed using the McNemara test. *p* values < 0.05 were considered statistically significant.

## 3. Results

### 3.1. Study Groups

The study groups differed significantly in age (*p* = 0.038) and sex distribution (*p* = 0.039). The mean age of patients after ICH was 67.3 years, while after IS it was 71.7 years. The group of patients after ICH was dominated by men—57.4%, while the group after IS was dominated by women—63.8%. The time of rehabilitation after stroke differed significantly between the groups (*p* < 0.001). Patients after ICH were rehabilitated approximately 29 days after the stroke incident, while patients after IS after 16 days after the stroke incident. After rehabilitation, patients after ICH achieved greater efficiency by 3 points on the NIHSS scale than after IS (*p* = 0.001). A detailed list of the study groups is presented in Table 1.

### 3.2. Cognitive Behavioral Disorders

In both groups of patients, there were significant mood disorders. After rehabilitation in 17% of patients after ICH and in as many as 40% of patients after IS, the depressed mood was observed (*p* = 0.007). Emotional, attention, and memory deficits dominated in both groups of the examined patients. Psychological therapy significantly reduced these deficits. The implemented therapy significantly reduced consciousness and orientation disorders in the group after ICH (*p* < 0.001). Moreover, in the group after ICH after rehabilitation, the speed of task performance in psychological tests significantly improved (*p* < 0.001). No significant improvement was noted in the remaining areas of cognitive-behavioral disorders. A detailed list is presented in Table 2.

### 3.3. Verbal Communication Disorders

There was no significant correlation between verbal communication disorders and the type of stroke before rehabilitation (*p* = 0.055) and after rehabilitation (*p* = 0.763). The use of speech therapy in both groups of patients significantly reduced disorders of speech articulation and aphasia. Moreover, in patients after ICH, rehabilitation significantly reduced language function deficits (*p* = 0.018). Before rehabilitation, over 59% of patients in both groups had proper interpersonal communication in terms of speech. In patients after ICH, the most common deficit was associated with the inadequate choice of words, while in patients after IS, the greatest problem was the formulation of sentences. The implemented therapy reduced disorders in 85% of patients after ICH (*p* = 0.001) and in 68% of patients after IS (*p* = 0.033). A detailed summary of the data is presented in Table 3.

### 3.4. Profile of Cognitive and Speech Therapy Disorders According to the ICF Classification Categories

**Figure 1:**
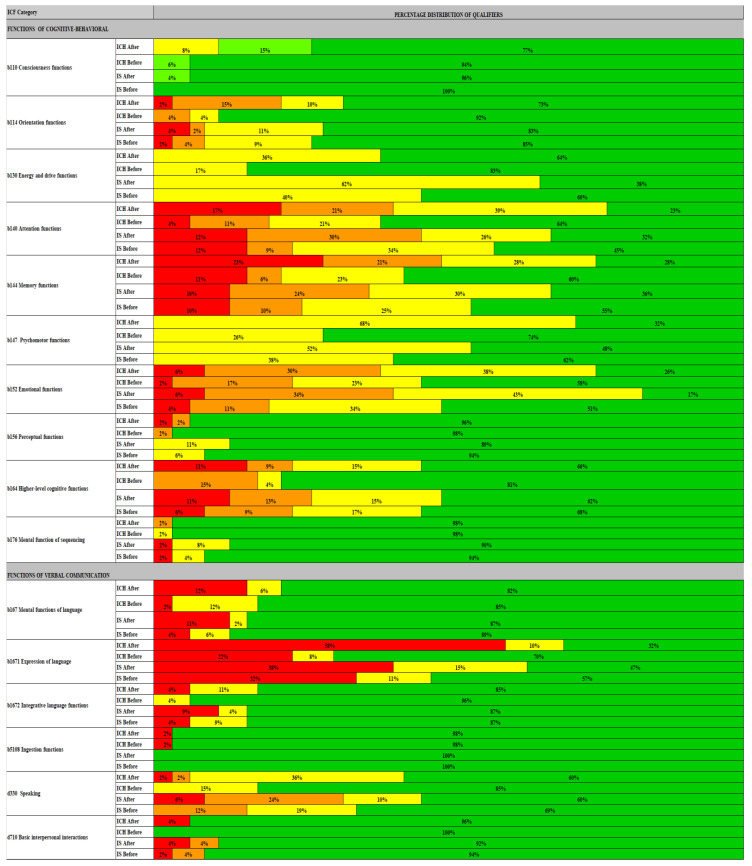
Profile of cognitive and speech therapy disorders according to the ICF classification categories. ICH—intracerebral hemorrhage; ICF—International Classification of Functioning, Disability and Health; IS—ischemic stroke; Red color—extremely high occurrence of disturbance; Orange color—significant occurrence of the disorder, Yellow color—moderate occurrence of the disorder; Light green color—significant occurrence of disturbance; Dark green color—no disorder present.

Figure 1 presents the ICF category sheet with the percentage distribution of the degree of impairment (extremely severe, moderate, slight, absent) of individual cognitive and speech therapy disorders depending on the history of IS or ICH and the condition before and after rehabilitation treatment.

#### 3.4.1. ICH

Before rehabilitation treatment in the group after ICH in the area of cognitive functions, the highest percentage of impairment defined as extremely high was observed in the following disorders: memory functions, attention functions and psychomotor functions. On the other hand, in speech therapy, the highest percentage of impairment was observed in mental functions and expression of language. At the level of significant impairment of functions were observed disorders in orientation and emotional functions. At the level of moderate functional impairment, disturbances in orientation, energy and drive functions, psychomotor functions and speech fluency were observed. As shown in Table 3, the implemented rehabilitation treatment resulted in the reduction of extensive significant and moderate disorders in the areas mentioned above in the evaluation after the rehabilitation treatment.

#### 3.4.2. IS

Before rehabilitation treatment in the group after ICH in the area of cognitive functions, the highest percentage of impairment defined as extremely high and also at a significant level was observed in the field of memory function disorders, attention systems, executive system and emotional function disorders. On the other hand, in speech therapy, the highest percentage of impairment was observed in disorders of linguistic functions and expression, as well as integrative language functions and fluency in speaking. At the level of moderate functional impairment, disturbances in energy and drive, psychomotor disturbances, and perceptual disturbances were observed. Targeted rehabilitation intervention in this case did not significantly reduce the degree of functional deficits. After rehabilitation treatment in the group after IS, there was no change in the category of dysfunction (extremely severe) or its change to a significant and moderate extent.

#### 3.4.3. ICH vs. IS

Before rehabilitation in the group after ICH, compared to the group after IS, a significantly higher percentage of impairment was observed at an extremely high level in regarding memory function disorders, attention systems, and expression of language. At the level of significant functional impairment, disturbances in memory functions and attention functions were observed more often. At the level of moderate functional impairment, psychomotor disturbances and fluency in speaking were observed more often. After rehabilitation treatment, a more significant reduction in functional impairment was observed in the group after ICH, from the extreme, severe and moderate degrees compared to the group after IS.

## 4. Discussion

Stroke is a significant social problem because it remains one of the main causes of morbidity and long-term disability and the second most frequent cause of death [10,11]. Therefore, a significant problem and, at the same time, a challenge in health policy of individual countries governments s is the coordination of activities aimed at reducing the degree of disability of stroke patients, and thus their re-activation in professional, social and family activities [13]. The research results presented in this study indicate that in the studied population, ICH was more common in people younger than IS (Table 1). This is in line with the observations of Yamada and colleagues [16]. The more frequent occurrence of ICH in men is confirmed in the studies by Zhang et al. [17] in relation to the European population. Perin et al. [18] conducted an interesting analysis of the influence of age on the detection of differences in the needs and goals of rehabilitation between older (>65 years) and younger (≤65 years) patients after stroke. The influence of gender on the effects of treating patients after stroke was also of interest to many researchers. Kim et al. [19] prove that women have more significant difficulties than men in recovering from the state of disability after acute stroke. Lai et al. [20] prove that poorer return to daily life activities and functioning in women after stroke compared to men may result from the older age of stroke, poorer physical fitness before stroke, and depression occurring more frequently after stroke. in turn, the latest reports by McDonald et al. [21] exclude a significant influence of gender on the functional status of patients after stroke. Important issue discussed in the literature is the dependence of the effects of early post-stroke rehabilitation on the type of stroke. As proved by Rost et al. [6] IS and ICH are the main sources of post-stroke cognitive impairment. According to the researchers, the type of stroke present is of key importance for developing of an individual rehabilitation and secondary prevention program. It is all the more important that in standard clinical practice, the rehabilitation program in the early period is methodologically very similar in both types of stroke [22,23]. As shown in Table 1, when analyzing the global NIHSS scale, the clinical type of stroke significantly determined the effects of rehabilitation. It should be clearly emphasized that after treatment, patients after ICH achieved greater efficiency than patients after IS. Similar to the conclusions of the presented study are the observations of Paoluci et al. [24] showing better effectiveness of rehabilitation treatment in the case of ICH than in the case of IS, despite the initially worse condition of patients after ICH. As evidenced by O’Donoghue et al. [25] in post-stroke therapy, multi-component interventions: motor rehabilitation combined with cognitive functions therapy have a beneficial effect on memory and other cognitive functions during rehabilitation.

Research by Hurfold et al. [26] shows that cognitive functions are most disturbed in the first month after ischemic stroke. In the group of patients studied in this study, regardless of the type of clinical stroke, the most frequent were emotional, attention and memory deficits (Figure 1). The obtained results are consistent with the research of Cumming et al. [27], who proved that stroke disturbs attention, memory and executive functions the most. According to Pater et al. [5], implementing psychological therapy reduces the incidence of cognitive disorders to 30%. Psychological therapy significantly reduced cognitive deficits in the studied groups of patients (Table 2). Moreover, in patients after ICH, the speed of task performance in psychological tests improved significantly. Additionally, Rasquin et al. [28] noticed that cognitive decline is a common after-stroke symptom manifested by slower information processing. On the other hand, Narasimahalu et al. [29] proved that the speed of information processing is of significant clinical importance in the prognosis of independence of patients after ischemic stroke. As Ruchinskas and Curyto [30] prove, the presence of depression may also influence the results of cognitive tests. It should be taken into account when treating cognitive disorders. Perhaps the result of the improvement in the function of the executive system (Table 2) in patients after a hemorrhagic stroke should be combined with the reduction of emotions in this group of patients after psychological therapy (Table 2). The obtained results are consistent with the research of Brodaty et al. [31], showed that after the treatment of cognitive dysfunctions, depression was maintained only in 27% of patients after ICH and 54% of patients after IS. On the other hand, Srikanth et al. [32] prove that the effectiveness of rehabilitation, apart from attention disorders and executive function disorders, is significantly influenced by verbal communication disorders. In the study, the use of speech therapy significantly reduced speech articulation and aphasia disorders as well as interpersonal communication disorders (Table 3). The obtained results are consistent with the research of Bhogal et al. [8], who proved that intensive speech therapy reduces speech articulation disorders and interpersonal communication. Additionally, Cicerone et al. [4] demonstrate that virtual therapy of cognitive functions reduces aphasia and apraxia in patients after stroke.

For practical reasons, the ICF classification includes as few categories as possible and at the same time, takes into account all categories needed to describe various aspects of the functioning of a patient with a specific disease in a comprehensive and cross-sectional assessment [33]. On the website https://www.icf-research-branch.org/ (accessed on 1 January 2020) WHO publishes examples of shortened functional assessment kits for stroke patients (Stroke Brief), for patients undergoing rehabilitation (Rehabilitation Set) and for neurological patients (Neurological Acute Brief) [34,35,36,37]. The profiles differ significantly in the selected assessment categories. In recent foreign publications, the authors correlate the ICF base code sets with the quality-of-life scales. [33,38] It is worth emphasizing that the ICF profile of functional assessment for patients in rehabilitation after stroke has not been published so far. The presented percentage distribution of cognitive-behavioral and verbal communication disorders based on the ICF qualifiers (Figure 1) presents, in one place, psychological and speech therapy disorders and the scale of their severity depending on the clinical type of stroke, which allows to make the right clinical decisions in during therapy planning. Additionally, Kohler et al. [39] prove that the ICF classification enables the organization of clinical data contained in medical records in a readable graphic form that allows for comparison and demonstration of differences.

## 5. Conclusions

(1). The type of stroke suffered determines the frequency and type of cognitive-behavioral and verbal communication disorders.
(2). The ICF classification can be used to assess the occurrence of psychological and speech therapy disorders depending on the clinical type of stroke.
(3). Using one tool during rehabilitation containing a description of cognitive-behavioral disorders as well as verbal communication in the form of an ICF sheet may improve communication between a speech therapist and a psychologist, which in turn may lead to an increase in the effectiveness of the therapy.

## Figures and Tables

**Table 1:** Characteristics of the studied groups.

		ICH	IS	*p*
Sex *n* (%)	Females	20 (42.6%)	30 (63.8%)	0.039 ^a^
	Males	27 (57.4%)	17 (36.2%)	
Age (years)	Mean ± SD	67.3 ± 10.6	71.7 ± 9.81	0.038 ^b^
	Median	66	70	
	Min–Max	49–92	51–92	
Time from stroke to rehabilitation (days)	Mean ± SD	33.5 ± 17.7	18.0 ± 8.6	<0.001 ^b^
	Median	29	16	
Duration of rehabilitation (days)	Mean ± SD	69.1 ± 32.2	59.0 ± 34.9	0.118 ^b^
	Median	63	51	
NIHSS before rehabilitation	Mean ± SD	13 ± 5	12 ± 4	0.847 ^b^
	Median	12	12	
	Min–Max	5–24	6–24	
NIHSS after rehabilitation	Mean ± SD	5 ± 5	9 ± 4	<0.001 ^b^
	Median	5	8	
	Min–Max	0–17	4–22	

^a^ chi2 test, ^b^ Mann–Whitney test, ICH—intracerebral hemorrhage; IS—ischemic stroke; *n*—size of the sample; NIHSS—National Institutes of Health Stroke Scale; *p*—statistical significance; SD—standard deviation.

**Table 2:** Consciousness and cognitive-behavioral disorders in patients after ICH and IS before and after rehabilitation.

ICF Category	Qualifier Criteria	ICH			IS			ICH vs. IS	
		Before	After	*p*	Before	After	*p*	Before	After
b110 Consciousness functions	0—full consciousness	36 (76.6%)	44 (93.6%)	0.005	45 (95.7%)	47 (100.0%)	1.0	0.007	0.784
	1—sleepy	7 (14.9%)	3 (6.4%)		2 (4.3%)	0 (0%)			
	2—not fully conscious	4 (8.5%)	0 (0%)		0 (0%)	0 (0%)			
	3—unconscious	0 (0%)	0 (0%)		0 (0%)	0 (0%)			
b114 Orientation functions	0—full orientation as to time and place preserved	34 (72.3%)	43 (91.5%)	0.002	39 (83.0%)	40 (85.1%)	0.180	0.201	0.343
	1—orientation to place and time preserved	5 (10.6%)	2(4.3%)		5 (10.6%)	4 (8.5%)			
	2—lack of full orientation as to the place or time	7 (14.9%)	2 (4.3%)		1 (2.1%)	2 (4.3%)			
	3—complete disorientation as to place and time	1 (2.1%)	-		2 (4.3%)	1 (2.1%)			
b130 Energy and drive functions	0—no mood disorders	17 (36.2%)	39 (83.0%)	<0.001	18 (38.3%)	28 (59.6%)	0.009	0.831	0.007
	1—depressed mood	30 (63.8%)	8 (17.0%)		29 (61.7%)	19 (40.4%)			
b140 Attention functions	0—no disturbances in attention systems	11 (23.4%)	30 (63.8%)	<0.001	15(31.9%)	21 (44.7%)	0.002	0.71	0.071
	1—slight attention disorders	18 (38.3%)	10 (21.3%)		12 (25.5%)	16 (34.0%)			
	2—moderate attention disorders	10 (21.3%)	5 (10.6%)		14 (29.8%)	4 (8.5%)			
	3-severe attention disorders	8 (17.0%)	2 (4.3%)		6 (12.8%)	6 (12.8%)			
b144 Memory functions	0—no memory impairment	13 (27.7%)	28 (59.6%)	<0.001	17 (36.2%)	26 (55.3%)	0.005	0.177	0.656
	1—mild memory impairment	13 (27.7%)	11 (23.4%)		14 (29.8%)	11 (23.4%)			
	2—moderate memory impairment	10 (21.3%)	3 (6.4%)		11 (23.4%)	5 (10.6%)			
	3—severe memory impairment	11 (23.4%)	5 (10.6%)		5 (10.6%)	5 (10.6%)			
b147 Psychomotor functions	0—no psychomotor slowing down	15 (31.9%)	35 (74.5%)	<0.001	23 (48.9%)	29 (61.7%)	0.074	0.06	0.184
	1—psychomotor slowing down	32 (68.1)	12 (25.5%)		24(51.06%)	18 (38.30%)			
b152 Emotional functions	0—no emotional disturbances	12 (25.5%)	27 (57.4%)	0.003	8 (17%)	24 (51.1%)	<0.001	0.464	0.725
	1—slight emotional disturbances	18 (38.3%)	11 (23.4%)		20 (42.6%)	16 (34.0%)			
	2—moderate emotional disturbances	14 (29.8%)	8 (17%)		16 (34.0%)	5 (10.6%)			
	3—severe emotional disturbances	3 (6.4%)	1 (2.1%)		3 (6.4%)	2 (4.3%)			
b156 Perceptual functions	0—no agnosia	45 (95.7%)	46 (97.9%)	0.18	42 (89.4%)	44 (93.6%)	0.18	0.281	0.331
	1—light agnosia	0 (0%)	0 (0%)		5 (10.6%)	3 (6.4%)			
	2—moderate agnosia	1 (2.1%)	1 (2.1%)		0 (0%)	0 (0%)			
	3—severe agnosia	1 (2.1%)	0 (0%)		0 (0%)	0 (0%)			
b164 Higher-level cognitive functions	0—no disorders of the executive systems	31 (66.0%)	38 (80.9%)	0.013	29 (61.7%)	32 (68.1%)	0.066	0.669	0.198
	1—slight disorders of the executive systems	7 (14.9%)	2 (4.3%)		7 (14.9%)	8 (17.0%)			
	2—moderate disorders of the executive systems	4 (8.5%)	7 (14.9%)		6 (12.8%)	4 (8.5%)			
	3—moderate disorders of the executive systems	5 (10.6%)	0 (0%)		5 (10.6%)	3 (6.4%)			
b176 Mental function of sequencing complex movements	0—no apraxia	46 (97.9%)	46 (97.9%)	1	42 (89.4%)	44 (93.6%)	0.583	0.101	0.309
	1—slight apraxia	0 (0%)	1 (2.1%)		4 (8.5%)	2 (4.3%)			
	2—moderate apraxia	1 (2.1%)	0 (0%)		0 (0%)	0 (0%)			
	3—severe apraxia	0 (0%)	0 (0%)		1 (2.1%)	1 (2.1%)			

Chi-squared test, ICH—intracerebral hemorrhage; IS—ischemic stroke; *p*—statistical significance.

**Table 3:** Verbal and social communication disorders in patients after ICH and IS before and after rehabilitation.

ICF Category	Qualifier Criteria	ICH			IS			ICH vs. IS	
		Before	After	*p*	Before	After	*p*	Before	After
b167 Mental functions of language	0—linguistic functions preserved	38 (80.9%)	40 (85.1%)	0.018	41 (87.2%)	42 (89.4%)	0.068	0.432	0.579
	1—partially impaired language functions	3 (6.4%)	6 (12.8%)		1 (2.1%)	3 (6.4%)			
	2—completely impaired linguistic functions	6 (12.8%)	1 (2.1%)		5 (10.6%)	2 (4.3%)			
b1671 Expression of language	0—no speech disorders	15 (31.9%)	33 (70.2%)	<0.001	22 (46.8%)	27 (57.4%)	0.028	0.075	0.193
	1—blurred speech	5 (10.6%)	4 (8.5%)		7 (14.9%)	5 (10.6%)			
	2—dysarthric speech	27 (57.4%)	10 (21.3%)		18 (38.3%)	15 (31.9%)			
b1672 Integrative language functions	0—no aphasia	40 (85.1%)	45 (95.7%)	0.003	41 (87.2%)	41 (87.2%)	0.012	0.845	0.133
	1—sensory or motor aphasia	5 (10.6%)	2 (4.3%)		2 (4.3%)	4 (8.5%)			
	2—sensory and motor aphasia	2 (4.3%)	0 (0%)		4 (8.5%)	2 (4.3%)			
b5108 Ingestion functions. other specified	0—no dysphagia	46 (97.9%)	46 (97.9%)	1	47(100.0%)	47(100.0%)	1	0.859	0.860
	1—dysphagia	1 (2.1%)	1 (2.1%)		0 (0%)	0 (0%)			
d330 Speaking	0—no word choice disorders	28 (59.6%)	40 (85.1%)	0.001	28 (59.6%)	32 (68.1%)	0.001	0.347	0.033
	1—inadequate. limited word choice	17 (36.2%)	7 (14.9%)		5 (10.6%)	9 (19.1%)			
	2—no sentence formulation	1 (2.1%)	0 (0%)		11 (23.4%)	6 (12.8%)			
	3—no speech. making sounds	1 (2.1%)	0 (0)%)		3 (6.4%)	0 (0%)			
d710 Basic interpersonal interactions	0—preserved	45 (95.7%)	47 (100%)	0.180	43 (91.5%)	44 (93.6%)	0.5	0.427	0.082
	1—moderately disturbed	0 (0%)	0 (0%)		2 (4.3%)	2 (4.3%)			
	2—completely disturbed	2 (4.3%)	0 (0%)		2 (4.3%)	1 (2.1%)			

Chi-squared test, ICH—intracerebral hemorrhage; IS—ischemic stroke; *p*—statistical significance.

## Data Availability

The data presented in this study are available on request from the first author. The data are not publicly available due to ethical restrictions.
